# The Entomopathogenic Fungus *Metarhizium anisopliae* Affects Feeding Preference of *Sogatella furcifera* and Its Potential Targets’ Identification

**DOI:** 10.3390/jof8050506

**Published:** 2022-05-15

**Authors:** Yirong Wang, Lijuan Han, Yuxian Xia, Jiaqin Xie

**Affiliations:** 1 School of Life Sciences, Genetic Engineering Research Center, Chongqing University, Chongqing 401331, China; w973077781@163.com (Y.W.); hljconan@163.com (L.H.); 2 Chongqing Engineering Research Center for Fungal Insecticides, and Key Laboratory of Gene Function and Regulation Technology under Chongqing Municipal Education Commission, Chongqing 401331, China

**Keywords:** entomopathogenic fungus, *Metarhizium anisopliae*, feeding behavior, *Sogatella furcifera*, olfactory-related genes, pest control

## Abstract

The rice planthopper *Sogatella furcifera* is a unique vector of the *southern rice black-streaked dwarf virus* (SRBSDV). The feeding behavior of *S. furcifera* should directly affect the diffusion of this virus. In this study, we noted that the infection of *Metarhizium anisopliae* CQMa421 on *S. furcifera* disturbed the feeding behavior of this pest to SRBSDV-infected rice, from preference to non-preference. Then, we further investigated the potential targets of *M. anisopliae* CQMa421 on the feeding behavior of *S. furcifera* after 0 h, 24 h and 48 h of infection by transcriptomic analysis via Illumina deep sequencing. A total of 93.27 GB of data was collected after sequencing, from which 91,125 unigenes were annotated, including 75 newly annotated genes. There were 1380 vs. 2187 and 137 vs. 106 upregulated and downregulated differentially expressed genes (DEGs) detected at 24 h and 48 h, respectively. The biological functions and associated metabolic processes of these genes were determined with the Gene Ontology (GO) and Kyoto Encyclopedia of Genes and Genomes (KEGG) databases. The results suggested that major of DEGs are involved in energy metabolism, biosynthesis, immune response, the FoxO signaling pathway, the MAPK signaling pathway and apoptosis in response to the fungal infection. Noteworthily, several olfactory-related genes, including odorant receptors and odorant binding proteins, were screened from these differentially expressed genes, which played critical roles in regulating the olfactory behavior of insects. Taken together, these results provide new insights for understanding the molecular mechanisms underlying fungus and host insect interaction, especially for olfactory behavior regulated by fungus.

## 1. Introduction

Rice is a staple crop, feeding more than half of the global population worldwide [1]. During the rice-growing period, it should face a large number of pest and disease threats, which seriously damage the quality and quantity of rice food [2,3]. The rice planthoppers, including *Sogatella furcifera*, *Nilaparvata lugens* and *Laodelphax striatellus*, are one of the most destructive pests in rice plants [2,4,5]. Those pests not only suck the sap out of rice straw to result in the death of rice plants, but also transmit several plant viruses, such as the *rice ragged stunt virus*, the *rice grassy stunt virus* and the *southern rice black-streaked dwarf virus* (SRBSDV) [3,6]. Moreover, these pests have strong reproductive and dispersal abilities, allowing them to damage the rice plants for the duration of the growing period. A previous study has reported that the occurrence of the rice virus SRBSDV keeps in line with the dispersal and outbreak of the *S. furcifera* population [7]. The damages caused by the plant virus is more severe than that caused by pests in some cases [8]. Thus, an effective method to control the proliferation of rice viruses is to block out the transmission of vectors (e.g., the rice planthoppers).

Entomopathogens commonly exist under natural conditions and play key roles in regulating the population of insects [9,10]. Compared to chemical insecticides, the fungal pesticides are environmentally friendly biocontrol agents with less risks for nontarget organisms [10]. Some entomopathogenic fungi, such as *Metarhizium anisopliae* and *Beauveria bassiana*, have been used for the control of agricultural and forestry pests, including *Chironomus riparius* [11], *Locusta migratoria* [12], *Helicoverpa armigera* [13], *Alphitobius diaperinus* [14] and *Nilaparvata lugens* [15,16]. Combined use of *M. anisopliae* and dsRNA or chemicals has shown good potential for the control of the rice pest *N. lugens* [16,17]. A specific *M. anisopliae* strain has also been applied for the control of resistant insect pests [18]. Moreover, industrial products of *M. anisopliae* have been reported that could suppress the population of rice planthoppers to low levels in large-scale applications [15].

Entomopathogenic fungi could directly penetrate the cuticles of insect pests by secreting a few proteases and chitinases under the function of turgor pressure [19]. After penetrating into the host body, rapid proliferation of fungal hyphal bodies in haemocoel would deprive the host of nutrients and result in its death [20]. Due to the immune response of the insect host, fungi would secrete several toxins to interfere with the host nervous system and suppress such immune responses, so as to benefit the reproduction of the hyphal body of the fungus [21,22]. Correspondingly, the number and type of host insect hemocytes would change to better interact with the invaded pathogens during the processes [23]. Meanwhile, the energy metabolism of the host is intensive, and many antimicrobial peptides are generated to function on the pathogens [24]. Although a few studies have examined the interaction between insect hosts and pathogens under different conditions by transcriptomic profiling [25,26,27], the means by which the rice planthopper *S. furcifera* responds to fungal infection is less known, especially in terms of their behaviors.

The olfactory behavior of insects is critical for feeding, mating, oviposition and avoiding natural enemies [28,29]. Those insects have evolved sophisticated, sensitive and specific olfactory systems to detect and discriminate amongst an enormous variety of odorants under complex environments [30]. They could identify special chemical odor molecules and locate the odor source by olfactory sensation and exhibit a behavioral response correspondingly [28,30]. For instance, the tobacco hawkmoths *Manduca sexta* preferentially oviposit on the jimson weed *Datura wrightii* due to the α-copaene induced via potato beetle *Lema daturaphila* infection [31]; the 4-vinylanisole is an important pheromone that could cause an aggregation behavior of the migratory locust *Locusta migratoria* [32]. The rice planthopper *S. furcifera* also shows a feeding preference for rice plants infected by the SRBSDV virus [33]. Generally, the olfactory behavior of insects is related to the expression level of certain genes, including the odorant binding proteins (OBPs), the odorant receptors (ORs) and the chemosensory proteins (CSPs) [28,34,35]. The knockdown or RNAi of those olfactory-related genes (e.g., OBPs, ORs or CSPs) would directly affect the feeding or oviposition behavior in insects [36].

The white-backed *S. furcifera* is a major vector of SRBSDV, and outbreaks of this pest may cause the prevalence of this virus [37]. Interestingly, this pest shows a preference to those rice plants that have been infected by SRBSDV, compared to non-SRBSDV-infected plants [33], which would benefit the transmission of this virus. Our previous study has reported that the fungal *M. anisopliae* CQMa421 could infect adults and nymphs of rice planthoppers [16,38]. However, whether infection of *M. anisopliae* would affect the behavior of *S. furcifera* is unknown. Thus, we first examined the feeding behavior of the rice pest *S. furcifera* after *M. anisopliae* infection. Transcriptomic profiling is a useful tool to investigate and screen for potential targets under different conditions [39,40]. To understand the potential mechanisms, and to seek the potential olfactory-related targets, we further studied the response of *S. furcifera* to *M. anisopliae* infection under different periods by transcriptomic analysis. The potential olfactory-related genes that might affect the feeding behavior of *S. furcifera* also have been screened from the DEGs. This study provides new insights for understanding the underlying mechanisms of an insect host interacting with a fungal infection, especially for the olfactory behavior regulated by the fungus. These results would improve the potential control strategies for the vector *S. furcifera* and its transmitted virus by integrated pest management.

## 2. Methods and Materials

### 2.1. Insect and Plant Culture

The rice planthopper *S. furcifera* used in this study was maintained and obtained from the Genetic Engineering Research Center, Chongqing University, Chongqing, China. *S.* The *furcifera* individuals were reared on rice seedings in cages (35 cm × 35 cm × 40 cm) at an insectary (T = 27 ± 1 °C; light:dark = 14 h:10 h). The SRBSDV rice plant was originally collected from a rice paddy in Nanxiong, Guangdong, China. To cultivate the SRBSDV rice plant, we first collected *S. furcifera* nymphs from SRBSDV rice plants to acquire this virus and then transferred them to a new normal (non-SRBSDV) rice plant in a tube for 2 or 3 days. In this way, the new normal rice plant would be infected by SRBSDV via *S. furcifera* nymphs. Prior to the next experiments, *S. furcifera* females and males were transferred to a cage (35 cm × 35 cm × 40 cm) containing new rice seedings for 48 h. We then collected the newly hatched nymphs for the feeding choice experiment and transcriptomic analysis.

### 2.2. Effects of M. anisopliae on S. furcifera Feeding Behavior

We collected *S. furcifera* adults after 5-day emergence for two feeding preference choices and random and Y-tube olfactory choice tests. In the random choice test, two differently treated rice plants (SRBSDV rice and non-SRBSDV rice plant) were transferred in a catercorner of flowerpots sealed with a net. Then, *S. furcifera* individuals were put in these flowerpots, and the number of insects on each rice plant was recorded at 3 h, 6 h, 18 h and 24 h. In the Y-tube olfactory choice experiment, we referenced the commonly used methods of a similar, previous study [41]. The number of *S. furcifera* in each arm of the Y-tube was recorded after staying more than 15 s. We then counted the total number of *S. furcifera* in each arm within 2 h.

The *M. anisopliae* CQMa421 strain (China General Microbiological Culture Collection Center, CGMCC No. 460) used in these experiments was obtained from the Genetic Engineering Research Center, Chongqing University, Chongqing, China. The fungal strain *M. anisopliae* CQMa421 was originally isolated from *Cnaphalocrocis medinalis* and stored in a −80 °C freezer in our laboratory. To evaluate the effects of *M. anisopliae* CQMa421 on the feeding behavior of *S. furcifera*, the *S. furcifera* adults were initially treated with 1 × 10^7^ conidia/mL suspension of *M. anisopliae*, which was prepared with the methods mentioned in our previous study [16]. Briefly, the *M. anisopliae* conidia were harvested after 15 days of growth in ¼ SDAY medium, which included 10 g glucose, 5 g yeast extract, 2.5 g peptone and 18 g agar/liter of sterilized water. Then, 1 × 10^7^ conidia/mL of *M. anisopliae* conidia was prepared using 0.1% Tween 80 by hemocytometer. A total of 1 mL of such conidial suspension was applied as a spray to the rice seedings by a Potter Precision Spray Tower (Burkard Manufacturing, UK). Sequentially, *S. furcifera* adults were transferred into the cage maintaining *M. anisopliae*-treated rice seedings and inoculated on them. Then, we used random choice and Y-tube olfactory choice to examine the feeding choice of *S. furcifera* for SRBSDV and non-SRBSDV rice plants at 48 h post-inoculation. The consequences of feeding choice in two tests were recorded according to the above methods.

### 2.3. Total RNA Isolation, Quantification and Sequencing

To further identify the potential olfactory-related targets, the *S. furcifera* individuals from the *M. anisopliae*-treated or control groups were collected at 0, 24 h and 48 h for transcriptomic analysis. Five sampling groups of *S. furcifera* were flash-frozen in liquid nitrogen and stored at −80 °C in a refrigerator prior to RNA extraction. Total RNA was extracted from whole *S. furcifera* individuals using TRIzol reagent (Invitrogen, Carlsbad, CA, USA). The purity, concentration and integrity of the RNA samples were tested using a NanoPhotometer^®^ spectrophotometer (IMPLEN, Westlake Village, CA, USA) and agarose gels to ensure the use of high-quality samples for transcriptome sequencing. Then, a total amount of 1 μg of RNA per sample was used for RNA sample preparation. Sequencing libraries were generated using a NEBNext UltraTM RNA Library Prep Kit for Illumina (NEB, Ipswich, MA, USA) following the manufacturer’s recommendations, and index codes were added to attribute sequences to each sample.

Briefly, mRNA was purified from total RNA using poly-T oligo-attached magnetic beads. Fragmentation was carried out using divalent cations under an elevated temperature in NEBNext First Strand Synthesis Reaction Buffer (5×). First-strand cDNA was synthesized using random hexamer primers and M-MuLV reverse transcriptase. Second-strand cDNA synthesis was subsequently obtained using DNA polymerase I and RNase H. The remaining overhangs were converted into blunt ends via exonuclease/polymerase activities. After adenylation of the 3′ ends of the DNA fragments, NEBNext adaptors with hairpin loop structures were ligated to prepare the samples for hybridization. To preferentially select cDNA fragments that were 240 bp in length, the library fragments were purified with the AMPure XP system (Beckman Coulter, Brea, CA, USA). Then, 3 μL of USER enzyme (NEB, Ipswich, MA, USA) was used with size-selected, adaptor-ligated cDNA at 37 °C for 15 min, followed by 5 min at 95 °C before PCR. Then, PCR was performed with Phusion High-Fidelity DNA polymerase, universal PCR primers and the index (X) primer. Finally, PCR products were purified by the AMPure XP system, and library quality was assessed on an Agilent Bioanalyzer 2100 system (Agilent, Santa Clara, CA, USA).

Clustering of the index-coded samples was performed on a cBot Cluster Generation System using a TruSeq PE Cluster Kit v4-cBot-HS (Illumina, San Diego, CA, USA), according to the manufacturer’s instructions. After cluster generation, the library preparations were sequenced on an Illumina platform and paired-end reads were generated.

### 2.4. Data Analysis

Raw data/raw reads in FASTQ format were first processed through in-house Perl scripts. In this step, clean data/clean reads were obtained by removing reads containing adapters, reads containing poly-N sequences and low-quality reads from the raw data. All downstream analyses were based on clean data with high quality. The adaptor sequences and low-quality sequence reads were removed from the data sets. At the same time, the Q20, Q30, GC content and sequence duplication level of the clean data were calculated. Raw sequences were transformed into clean reads after data processing. These clean reads were then mapped to the reference genome sequence [42]. Only reads with a perfect match or one mismatch were further analyzed and annotated based on the reference genome. Hisat2 software was used to map reads to the reference genome. The Illumina sequence reads have been submitted to the NCBI SRA database (accession No. PRJNA786731).

Gene functional annotation was based on the following databases: Nr (NCBI nonredundant protein sequences); Pfam (protein family); Nt (NCBI nonredundant nucleotide sequences); KO (KEGG orthologue database); SWISS-PROT (a manually annotated and reviewed protein sequence database); and GO (gene ontology). Gene expression levels were estimated by the fragments per kilobase of transcript per million fragments mapped (FPKM) values, with the following formula:

FPKM = cDNA Fragments/Mapped Fragments (Millions) * Transcript Length (kb)

Differential expression analysis was performed using DESeq2, which provided statistical analyses for determining differential expression in digital gene expression (DGE) data using a model based on the negative binomial distribution. The resulting *p* values were adjusted using Benjamini and Hochberg’s approach for controlling the false discovery rate. Genes with an adjusted *p* value < 0.01 found by DESeq2 were considered to be differentially expressed. Gene ontology (GO) enrichment analysis of the differentially expressed genes (DEGs) was implemented by the GOseq R package based on the Wallenius noncentral hypergeometric distribution, which adjusts for gene length bias in DEGs. KEGG is a database resource for understanding the high-level functions and utilities of biological systems, such as cells, organisms, and ecosystems, from molecular information, especially large-scale molecular datasets generated by genome sequencing and other high-throughput experimental technology (http://www.genome.jp/kegg/, 17 November 2021). KOBAS software was then used to test the statistical enrichment of DEGs in KEGG pathways.

### 2.5. Validation of DEGs Library and Olfactory-Related Genes Identification

To validate the DEGs in the libraries, 20 DEGs (i.e., control vs. treatment) were randomly selected for a comparison using real-time quantitative PCR (qPCR). Moreover, the olfactory-related genes (e.g., ORs, OBPs and CSPs) in DEGs were also screened from the DEGs and analyzed by qPCR. The qPCR was performed on an iCycler iQ real-time PCR system (Bio-Rad, Hercules, CA, USA) with a QuantiNova SYBR Green PCR Kit (QIAGEN, Dusseldorf, Germany) according to the manufacturer’s instructions. The cycling parameters were as follows: initial denaturation at 94 °C for 30 s, followed by 40 cycles of 94 °C for 10 s, 55 °C for 30 s and 95°C for 1 s. The β-actin was selected as the reference gene for the normalization of the expression of the DEGs, according to the 2^−ΔΔCt^ method. The primers designed for qPCR in this experiment are listed in Table S1.

## 3. Results

### 3.1. Effects of M. anisopliae on Feeding Choice of S. furcifera

**Figure 1:**
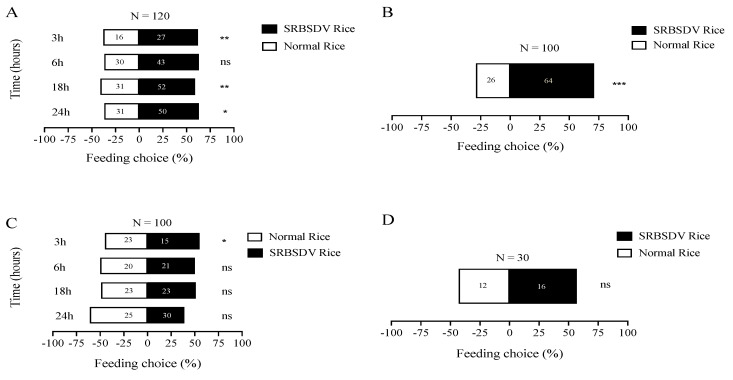
The effects of *M. anisopliae* on host *S. furcifera* feeding behavior. (**A**) Feeding choice of *S. furcifera* for SRBSDV or non-SRBSDV rice plants under random feeding test; (**B**) feeding choice of *S. furcifera* for SRBSDV or non-SRBSDV rice plants under Y-tube olfactory test; (**C**) after 48 h infection, the feeding choice of *S. furcifera* for SRBSDV or non-SRBSDV rice plants under random feeding test; (**D**) after 48 h infection, the feeding choice of *S. furcifera* for SRBSDV or non-SRBSDV rice plants under the Y-tube olfactory test. The asterisks indicate the significant difference. “*” indicates *p* < 0.05; “**” indicates *p* < 0.01 and “***” indicates *p* < 0.001.

We first checked the feeding behavior of *S. furcifera* on rice plants infected with SRBSDV or non-SRBSDV by random choice and Y-tube olfactory choice tests. In the random feeding choice test, the pest *S. furcifera* showed a feeding preference for the rice plant infected with SRBSDV during 24 h, except the time at 6 h (Figure 1A). Similarly, we also noted that this pest exhibited a feeding preference for the arm of the Y-tube connected with the SRBSDV-treated rice plant (Figure 1B, *p* < 0.001; *t*-test). However, when the pests were challenged by fungal *M. anisopliae* CQMa421, they showed no apparent feeding preference for any of the two rice plants (i.e., SRBSDV or non-SRBSDV rice plants), both in the random (except 3 h; see Figure 1C) and the Y-tube olfactory choice tests (Figure 1D).

### 3.2. Summary of Digital Gene Expression by Transcriptomic Profiling

To investigate potential targets affecting the feeding preference of the rice planthopper *S. furcifera* induced by the fungal *M. anisopliae* challenge, we further conducted the transcriptomic responses of *S. furcifera* to *M. anisopliae* inoculation after 0 h, 24 h and 48 h. A total of 15 DGE tag libraries were constructed, including the T-24 h vs. blank (0 h), T-24 h vs. CK-24 h, T-48 h vs. blank (0 h) and T-48 h vs. CK-48 h comparison for different treatments. By Illumina deep sequencing, 38.39~46.64 million raw reads were obtained from each treatment sample (Table 1). Prior to mapping, low-quality and adapter reads were filtered and 35.83~44.91 million clean-sequence reads per library were retained (Table 1). All samples had Q30 values greater than 92%, and the GC content ranged from 39.42% to 42.44% (Table 1), suggesting the reliability of the sequencing results.

### 3.3. Transcriptomic Comparison and Analysis of M. anisopliae Inoculation

**Figure 2:**
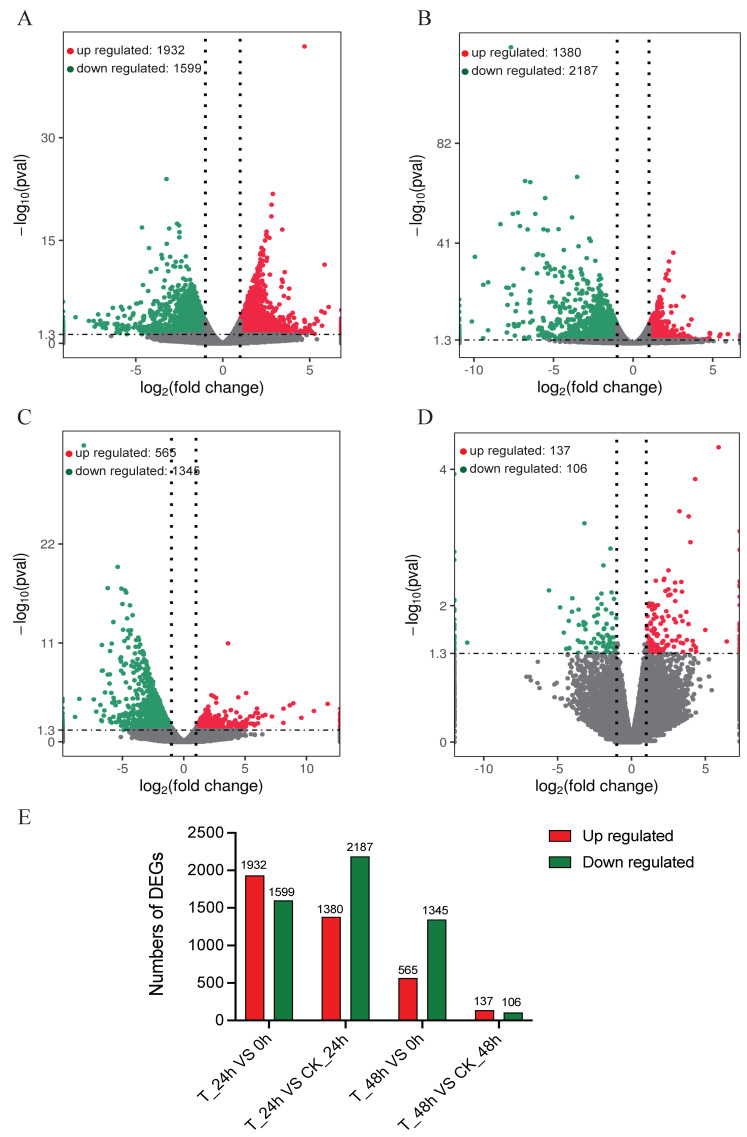
The volcano plot and numbers of differentially expressed genes (DEGs) of *S. furcifera* identified after *M. anisopliae* inoculation. (**A**) The volcano plot of DEGs at 24 h post inoculation vs. 0 h; (**B**) the volcano plot of DEGs at 24 h post inoculation vs. 24 h control; (**C**) the volcano plot of DEGs at 48 h post inoculation vs. 0 h; (**D**) the volcano plot of DEGs at 48 h post inoculation vs. 48 h control; and (**E**) the number of *S. furcifera* DEGs during *M. anisopliae* infection from 0 to 48 h.

To compare the DEGs among 15 different libraries, the gene expression levels were first determined from the FPKM values. Global analysis of the transcriptomic changes in *S. furcifera* at different time points after *M. anisopliae* inoculation demonstrated up- or downregulated genes in the control and treatment groups. DESeq.2 was selected to test DEGs with *p* < 0.01. These results showed that 1932 genes were upregulated at 24 h after fungal inoculation, and 1599 genes were downregulated by over two folds (|log2 (FoldChange)| > 2) in T-24 h vs. 0 h (Figure 2A). These results showed that 1380 genes were upregulated at 24 h after fungal inoculation, and 2187 genes were downregulated by over two folds (|log2 (FoldChange)| > 2) in T-24 h vs. CK-24 h (Figure 2B). At 48 h, there were 565 upregulated and 1345 downregulated genes (Figure 2C) in T-48 h vs. 0 h. By contrast, there were 137 upregulated and 106 downregulated genes after 48 h inoculation in T-48 h vs. CK-48 h (Figure 2D). These results showed that many different genes would be involved in the *S. furcifera* response to *M. anisopliae* CQMa421 infection over time (Figure 2E). We also found 1051, 44, 929 and 54 DEGs commonly expressed for T-24 h vs. CK-24 h and T-24 h vs. 0 h, for T-24 h vs. CK-24 h and T-48 h vs. CK-48 h, for T-24 h vs. 0 h and T-48 h vs. 0 h and for T-48 h vs. CK-48 h and T-48 h vs. CK-0 h, respectively (Figure S1). Specifically, the common 44 DEGs of the 24 h and 48 h treatment vs. control were majorly involved in RNA-directed DNA polymerase (g17653, g22334, g36185 and g48810), fatty acyl-CoA reductase (g82369), sphingosine kinase (g36165), glycerol-3-phosphate dehydrogenase (g14910) and malate dehydrogenase (g86484) (Table S2).

### 3.4. Functional Classification and Pathway Analysis

**Figure 3:**
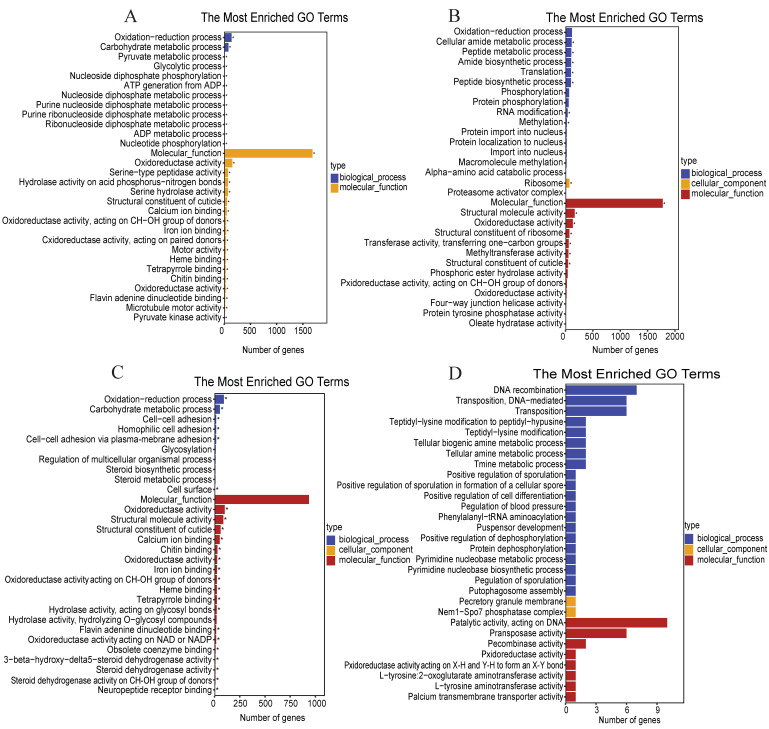
Gene ontology (GO) categories of *S. furcifera* DEGs after challenge with *M. anisopliae* at different periods. (**A**) GO categories of *S. furcifera* DEGs after challenge with *M. anisopliae* at 24 h vs. 0 h; (**B**) GO categories of *S. furcifera* DEGs after challenge with *M. anisopliae* at 24 h; (**C**) GO categories of *S. furcifera* DEGs after challenge with *M. anisopliae* at 48 h vs. 0 h; and (**D**) GO categories of *S. furcifera* DEGs after challenge with *M. anisopliae* at 48 h. The asterisks (*) indicate the significant enrichment of GO terms.

To examine the functions of the DEGs after *M. anisopliae* inoculation, we used the GO databases to map them. Specifically, after 24 h inoculation, the biological function category was assigned to 2008 DEGs (Figure 3A) compared with 0 h, and the biological function category was assigned to 2084 DEGs with the control after 24 h (Figure 3B). By contrast, the biological function category was assigned to 1107 DEGs (Figure 3C) after 48 h, compared with 0 h. However, the biological function category included 101 DEGs after 48 h with the control treatment, indicating different physiological responses of host *S. furcifera* to fungal infection over time (Figure 3D). Totally, the annotated GO terms included 5300 DEGs (2008, 2084, 1107 and 101 DEGs) in the BLAST database within the categories of biological process, cell component and molecular function (Figure 3A–D). For different periods, it also showed different abundance among the categories, and most of the DEGs were enriched in energy metabolism and antimicrobial activity during this period (Figure 3B,D).

**Figure 4:**
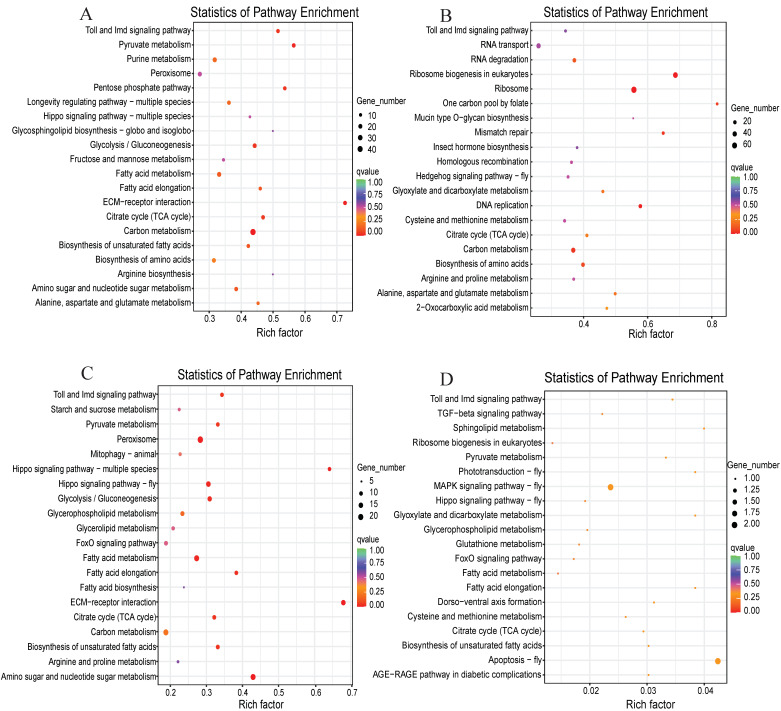
Enrichment and dispersion point map of differentially expressed genes (DEGs) in KEGG pathways. (**A**) The DEGs involved in the pathways after challenge for 24 h vs. 0 h; (**B**) the DEGs involved in the pathways after challenge for 24 h; (**C**) the DEGs involved in the pathways after challenge for 48 h vs. 0 h; (**D**) the DEGs involved in the pathways after challenge for 48 h. The circles in the graph indicate that the KEGG pathway with the gene number and enrichment factor (q-value, different colors) are displayed on the y and x axes, respectively.

In this study, we also selected KEGG to identify the metabolic and signal transduction pathways associated with these DEGs. The DEGs were mapped to 133 pathways in the KEGG database between the blank control (0 h) and treatment groups at 24 h inoculation (Figure 4A), and 110 pathways were mapped in the KEGG database between the control and treatment groups at 24 h post-inoculation (Figure 4B). By contrast, the DEGs between the blank control (0 h) and treatment groups at 48 h post-inoculation were mapped to 107 pathways in the KEGG database (Figure 4C), and the DEGs between the control and treatment groups at 48 h post-inoculation were mapped to 25 pathways in the KEGG database (Figure 4D). The top 20 pathways of each group in the richness analysis were exhibited in Figure 4, majorly including the pathways of carbon metabolism at 24 h vs. 0 h and the pathways of ribosome and its biogenesis at 24 h vs. control. Meanwhile, we noted that many pathways were involved in the peroxisome, amino sugar and nucleotide sugar metabolism at 48 h vs. 0 h, and in apoptosis and the MAPK signaling pathway at 48 h vs. control. These pathways are important for maintaining the physiological functions of the host insect and for enhancing its response to pathogenic invasion.

From the top 20 highly or lowly DEGs in host *S. furcifera* after *M. anisopliae* infection, the identified DEGs by the Nr annotation were mostly included in the cuticular proteins (g53545 and g51851), the nose resistant to fluoxetine protein 6 (g39720) and the elongation of the very long chain fatty acids protein (g80315). The upregulated targets were involved in retrovirus-related Pol polyprotein (g39683) and RNA-directed DNA polymerase from mobile element jockey (g36211 and g23323). After 24 h inoculation, the vitellogenin (g89906, g89903 and g89900) and serine protease (g89852) were downregulated, but the RNA-directed DNA polymerase (g31405 and g87480) was upregulated. We also noted that some of the targets, including the cardioaccelerator peptide receptor (g56305) and the nucleic-acid-binding protein (g48675) were downregulated after 48 h inoculation. In contrast, the putative RNA-directed DNA polymerase (g57847), adult-specific cuticular protein (g71543) and dopamine receptor (g88013) were upregulated during this period (Table S3).

### 3.5. Validation of DEGs Using RT-qPCR and Olfactory-Related Genes Identification

**Figure 5:**
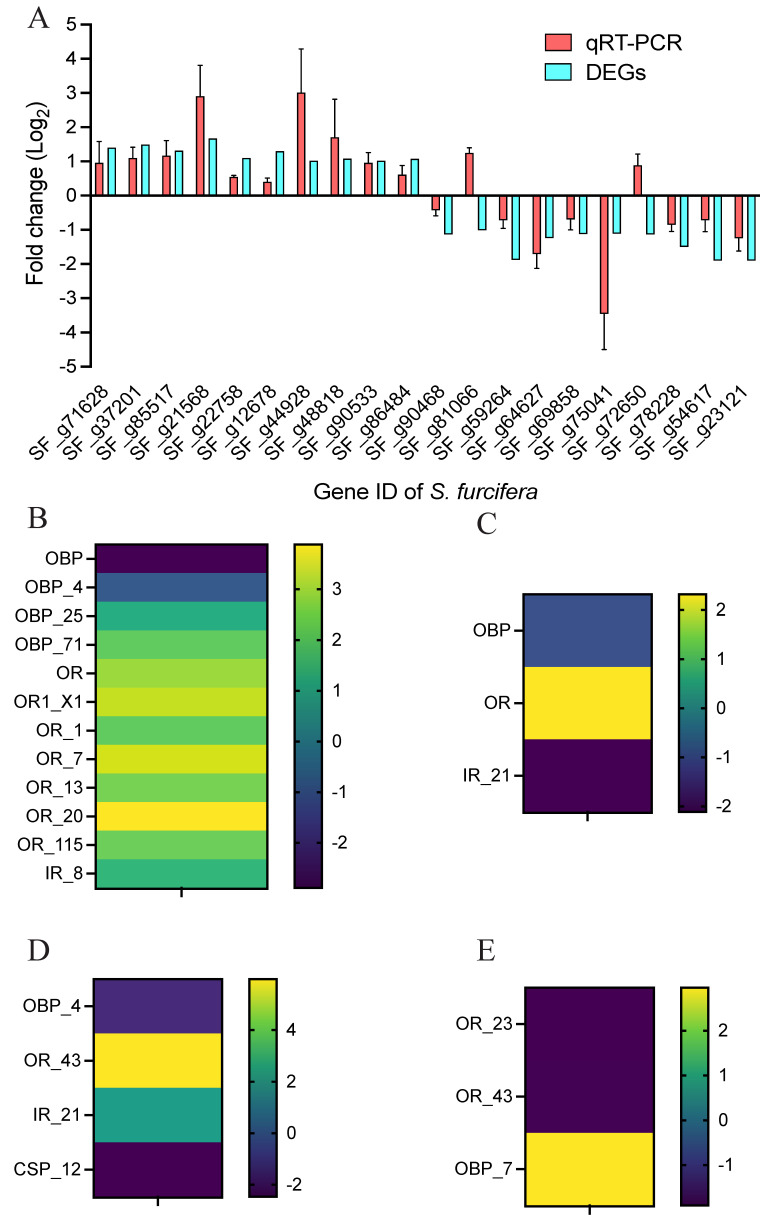
Transcriptomic validation by qPCR and the olfactory-related genes identification. (**A**) A comparison of qPCR and RNA-seq of 20 genes; (**B**) the DEGs involved in the regulation of host olfactory behavior at 24 h vs. 0 h; (**C**) the DEGs involved in the regulation of host olfactory behavior at 24 h; (**D**) the DEGs involved in the regulation of host olfactory behavior at 48 h vs. 0 h; and (**E**), the DEGs involved in the regulation of host olfactory behavior at 48 h.

To further validate the RNAs identified through sequencing, we used qPCR to evaluate the results of RNA sequencing. We selected 20 of the highly or lowly expressed DEGs based on the Illumina sequencing results. The results showed that the expression trends of the selected RNAs showed a slight discrepancy from the findings of the sequencing analysis (Figure 5A), which might be due to the differences in the sensitivity, specificity and applied algorithms between the two techniques.

The expression level of olfactory-related genes plays key roles in regulating the feeding behavior of insects. Thus, we further examined the potential genes that might affect the feeding preference of *S. furcifera* after *M. anisopliae* infection. We screened a few olfactory-related genes, including OPBs, ORs, IRs and CSPs, from the DEGs after *M. anisopliae* inoculation (Figure 5B–E). Interestingly, we noted that the OR_23 and OR_43 were downregulated at 48 h post-inoculation, which may be potential target genes affecting the feeding behavior of *S. furcifera*.

## 4. Discussion

Damage by insect pests, including rice planthoppers, *Cnaphalocrocis medinalis* and *Chilo suppressalis*, are one of major factors resulting in losses of rice yields [2,43]. The population of rice planthoppers has evolved a high resistance to many commonly used insecticides [44]. On the other hand, those pests are important vectors of rice plant viruses (e.g., the *rice ragged stunt virus*, the *rice grassy stunt virus* and the *southern rice black-streaked dwarf virus*) for indirectly affecting the growth of rice plants further [45]. Previous studies have reported that the olfactory behavior of insect vectors would affect the efficiency of virus transmission and dispersal [46,47]. Moreover, carrying plant viruses could regulate the feeding behavior of insect vectors [33,48]. Thus, avoiding or disturbing the feeding behavior of vectors is a potential strategy to suppress the plant virus. In this study, we noted that the feeding preference of *S. furcifera* for SRBSDV rice plants has changed after fungal *M. anisopliae* infection. A further transcriptomic analysis suggests that the infection of *M. anisopliae* on *S. furcifera* would affect the expression of many potential targets, including several olfactory-related genes.

The rice virus SRBSDV is a serious plant virus, which could result in considerable yield losses of rice [6]. After its first report in Goungdong, China in 2008, the virus has spread into Japan and Vietnam, and has become one of the most important plant viruses for rice [37]. The rice planthopper *S. furcifera* is the unique vector of SRBSDV with a long-life transmitting ability. The occurrence of SRBSDV is related to the breakout of the *S. furcifera* population under field conditions [7]. Interestingly, this pest showed a feeding preference for those rice plants infected with SRBSDV, due to some specific odors [39]. Another insect pest, the whitefly, also showed a steady preference for *Tomato yellow leaf curl virus*-infected plants [49]. The preference of vectors would enhance the probability of the virus to enter uninfected hosts, and eventually benefit the virus’ spread among the plant community [49,50]. However, we found that the feeding preference of *S. furcifera* for certain target plants disappeared at 48 h post-inoculation.

Transcriptome profiling is a beneficial method to identify the potential genes that regulate physiology, growth and behavior. In this study, to understand how the *M. anisopliae* regulated the feeding behavior of *S. furcifera*, we identified the DEGs after fungal inoculation at 0 h, 24 h and 48 h. During fungal infection, a series of metabolic pathways are screened, including amide and peptide biosynthesis, carbohydrate catabolic processes and immune responses (GO0002429, GO0002757 and GO0002764, respectively). In fact, the recognition of hosts by pathogens occurred in the initial inoculation stage within 4 h. We also noted the cell surface receptor signaling pathway (GO0002768) in *S. furcirera* after 24 h inoculation. The recognition molecules, such as peptidoglycan recognition proteins (PGRPs), β-1,3- glucan recognition proteins (βGRPs), galectins, C-type lectins (CTLs) and scavenger receptors (SCRs) [24,27,51], play vital roles during this period. However, we did not investigate the initial stages after this fungal inoculation in this study, investigating the stages after 24 h instead. The high-expression genes are majorly involved in RNA-directed DNA polymerase (g36211, g31405 and g87480) during this period. In contrast, the suppressed metabolisms are involved in serine protease (g89852) and farnesol dehydrogenase (g28615). Moreover, the MAPK signaling pathway (dnx04013), apoptosis (dnx04214), Toll and IMD signaling pathway (dnx04624) and FoxO signaling pathway (dnx04068) were activated at 48 h post-inoculation.

Importantly, we identified several DEGs of olfactory-related genes after the fungal *M. anisopliae* inoculation under different periods. The olfactory behaviors of insects are important clues for targeting foods, mating and avoiding natural enemies [28,29]. The oviposition behavior was disturbed after the Or35 deficiency in the tobacco hawkmoths *Manduca sexta* [31] and the Or31 deficiency in *Helicoverpa assulta* [52]. In this study, we noted that the feeding preferences have been disturbed, from preference to non-preference for SRBSDV rice plants, after *M. anisopliae* infection. This phenomenon indicated that *M. anisopliae* might affect the olfactory capability by interacting with these targets. Indeed, we found several differentially expressed OBPs and ORs after *M. anisopliae* infection. An altered expression of CSPs and OBPs in response to fungal infection has also been reported in the red fire ant *Solenopsis invicta* [53]. Those genes play important roles in regulating the host behavior, especially feeding behaviors. Moreover, feeding behaviors would affect the transmission of rice viruses due to their dependency on the insect vector [33,54]. The variation of feeding preferences to targeting rice plants would have a further impact on the dispersal of SRBSDV from plant to plant. Thus, we may further develop strategies (e.g., RNAi to target OR OBP) to regulate the efficiency of vector *S. furcifera* transmitting this virus.

In conclusion, we noted that the infection of *M. anisopliae* could disturb the feeding behavior of host *S. furcifera* and further identified several olfactory-related genes (i.e., ORs and OBPs) by transcriptomic profiling. These results not only indicate that the fungal *M. anisopliae* can regulate host behavior, but also suggest such regulations may be involved in the expression level of olfactory-related genes. Future work will warrant the specific functions of several ORs and OBPs, and develop effective approaches for the control of this pest and its transmitted SRBSDV.

## Figures and Tables

**Table 1:** Statistical summary of *S. furcifera* groups, after having inoculated *M. anisopliae* or not.

Sample Name	Raw Reads	Raw Bases	Clean Reads	Clean Bases	Q30
T_24 h_1	38,699,940	5.80G	35,830,030	5.37G	92.91%
T_24 h_2	42,519,476	6.37G	40,606,948	6.09G	93.49%
T_24 h_3	38,627,148	5.79G	36,601,008	5.49G	93.25%
CK_24 h_1	41,683,106	6.25G	39,847,928	5.98G	93.70%
CK_24 h_2	44,749,802	6.71G	42,760,666	6.41G	93.92%
CK_24 h_3	46,649,430	6.99G	44,910,818	6.74G	93.08%
T_48 h_1	40,096,262	6.01G	38,405,376	5.76G	93.02%
T_48 h_2	42,917,082	6.43G	41,055,774	6.16G	93.73%
T_48 h_3	40,289,444	6.04G	38,627,478	5.79G	93.26%
CK_48 h_1	43,887,028	6.58G	42,403,914	6.36G	92.82%
CK_48 h_2	38,657,406	5.79G	36,866,622	5.53G	93.27%
CK_48 h_3	40,229,530	6.03G	38,382,242	5.76G	93.54%
Blank_1	41,866,614	6.27G	40,000,444	6.00G	93.20%
Blank_2	38,396,532	5.75G	36,692,418	5.50G	94.56%
Blank_3	43,095,904	6.46G	41,381,568	6.21G	94.07%

T, *M. anisopliae* treatment group; CK, control group.

## Data Availability

All sequence data are available in NCBI GenBank following the accession number No. PRJNA786731 in the manuscript.

## Supplementary Materials

The following are available online at https://www.mdpi.com/article/10.3390/jof8050506/s1, Figure S1: The Venn diagram of DEGs after the fungal infection. Table S1: The primers used for RT-qPCR in this study. Table S2: The descriptions of commonly expressed DEGs in Venn diagram after *M. anisopliae* infection. Table S3: Top 20 highly or lowly DEGs in different period of *M. anisopliae* infection.
