# Supplementary material for: The Entomopathogenic Fungus Metarhizium anisopliae Affects Feeding Preference of Sogatella furcifera and Its Potential Targets’ Identification
Source: J Fungi (Basel). 2022 May 15;8(5):506. doi: 10.3390/jof8050506 (PMC9147605; doi:10.3390/jof8050506)
Supplement: Supplementary file 1 [file jof-08-00506-s001.zip › jof-1719784-supplementary.pdf]

Figure S1. The Venn diagram of DEGs after the fungal infection.

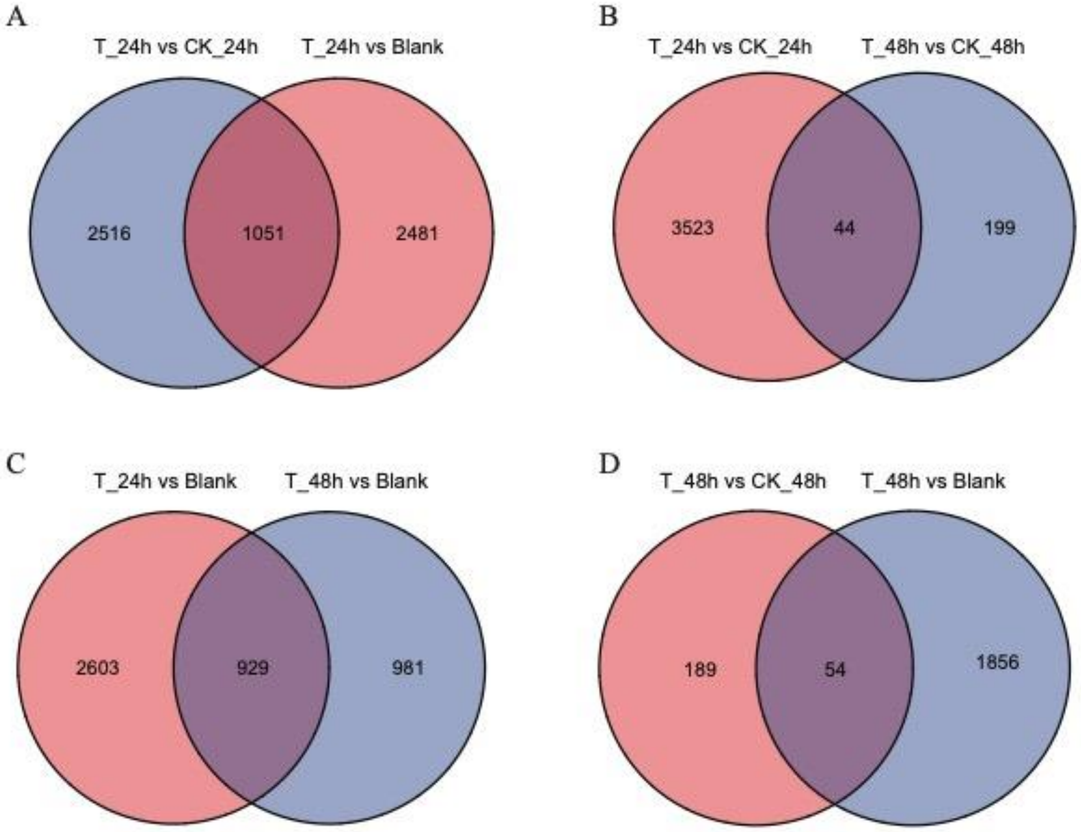

The Venn diagram of DEGs after the fungal infection at 0, 24h and 48h. A, the number of DEGs in Venn diagram of T-24h vs CK-24h and T-24h vs 0h; B, the number of DEGs in Venn diagram of T-24h vs CK-24h and T-48h vs CK-48h; C, the number of DEGs in Venn diagram of T-24h vs 0h and T-48h vs 0h; D, the number of DEGs in Venn diagram of T-48h vs CK-48h and T-48h vs CK-0h.

Table S1. The primers used for RT-qPCR in this study.

Gene ID	Primer directions	Primer sequences (5'-3')
SF_71628	Forward	GACGAGGAGTTCACCCTGGA
	Reverse	TTGGTGAGCGATGCCAGTTC
SF_37201	Forward	TCCTATGACATCACAACA
	Reverse	GACCAGAACAGTAACTAAC
SF_85517	Forward	GTGGTTCCCAATGCTCAGGG
	Reverse	TGCCAGATCCATGTTCAGCC
SF_21568	Forward	GCTACACGTCAGGCACAACA
	Reverse	GTACATCCCGTTCTCGCAGC
SF_22758	Forward	TGGAGGTTACGGCAGAGCTT
	Reverse	CCAGGTAGCCTCCTTCACCA
SF_12678	Forward	GAGGCTACTCGTGGGTGGAT
	Reverse	CGCAGAGGTAGTCGCAGTTG
SF_44928	Forward	GCTCAGATGGTGGGATTGGC
	Reverse	TTGGCAAGCTGATGCTGGAC
SF_48818	Forward	CATTCCTGAGAGAGTAGT
	Reverse	TTACCTATTGAAGAGAACAC
SF_90533	Forward	GTCACGACGCCTCCAAAGTC
	Reverse	CCGAGTGAGGCTTGTCGTTC
SF_86484	Forward	AAGTAGTGAAGGCTAAGG
	Reverse	CTGGAGTGGAGAAGTATT
SF_90468	Forward	CTGCGGTACAGGGTCTGGTA
	Reverse	ACTTGTACTGTCCCGCTCCA
SF_81066	Forward	TGACACCCACCTCACACGAT
	Reverse	AGCGCACAGTATCTCCGAGT
SF_59264	Forward	GACGAGGAGTTCACCCTGGA
	Reverse	TTGGTGAGCGATGCCAGTTC
SF_64627	Forward	GCACACCGTCCAGTTCTACG
	Reverse	ACAGCCAGCGTGATATTGGC
SF_69858	Forward	GTGGTTGTCTCTGTCTAC
	Reverse	ACGGTGTGAAGGATATTG
SF_75041	Forward	ATGCAGGACGACTGGAGACA
	Reverse	ACGCTCGTACTGCTCCTGAT
SF_78228	Forward	ATACCAGGCTCAGGCGACAT
	Reverse	GAAAGTGCGGCGTTAGTGGA

SF_72650	Forward	CCACAGTTCAGGCCGTTCAG
	Reverse	CTTGTCGCTCACGCAGTTGA
SF_54617	Forward	TCCCGATCACGAGTCCTTGG
	Reverse	TTTGACAGAGCCCAGACCCA
SF_23121	Forward	ACGCCAGCAGCAGGATTTC
	Reverse	GCCAACTCCCTTTCAGCACTA
SF_β-actin	Forward	GTGACTTGACCGACTACC
	Reverse	TCTCCTTGATGTCCCTGA

Table S2. The descriptions of commonly expressed DEGs in Venn diagram of 24h vs 48h after *M. anisopliae* infection.

Gene ID	FoldChange (Log2)	P-value	NR_Description	KOG_Description
Novel00528	2.5	1.75E-03	LOC100569743 [*Acyrthosiphon pisum*]	-
Novel00733	1.26	1.54E-03	-	-
g14910	1.59	4.12E-13	Glycerol-3-phosphate dehydrogenase, mitochondrial isoform X2 [*Polistes canadensis*]	Glycerol-3-phosphate dehydrogenase
g16571	1.18	2.88E-02	LOC100889621 [*Strongylocentrotus purpuratus*]	FOG: Reverse transcriptase
g17653	1.73	1.96E-03	RNA-directed DNA polymerase from transposon BS [*Exaiptasia pallida*]	-
g20931	1.07	2.78E-03	Homeobox protein ARX [*Camponotus floridanus*]	Transcription factor, contains HOX domain
g21429	2.25	3.44E-34	Troponin C, isoform 1 [*Trachymyrmex cornetzi*]	Calmodulin and related proteins (EF-Hand superfamily)
g22334	1.48	5.28E-07	Putative RNA-directed DNA polymerase from transposon BS, partial [*Exaiptasia pallida*]	FOG: Reverse transcriptase
g25610	1.05	3.21E-02	-	-
g25755	-1.66	3.16E-02	TPA_inf: HDC03386 [*Drosophila melanogaster*]	-
g27842	4.83	1.65E-04	-	-
g28303	-3.9	5.33E-03	-	-
g30040	1.56	2.71E-08	-	-
g30622	2.56	7.76E-06	-	-

g30624	2.25	3.62E-03	Down syndrome cell adhesion molecule-like protein Dscam2 isoform X2 [*Drosophila kikkawai*]	-
g36165	-1.24	4.10E-02	Sphingosine kinase 1 [*Dinoponera quadriceps*]	Sphingosine kinase, involved in sphingolipid metabolism
g36183	1.89	8.02E-03	RNA-directed DNA polymerase from transposon BS [*Exaiptasia pallida*]	FOG: Reverse transcriptase
g37069	1.63	3.50E-03	LOC100158863 [*Acyrthosiphon pisum*]	-
g38088	-1.21	1.27E-02	-	-
g41009	-2.44	4.09E-15	Tumor protein p63 isoform alpha 2-like isoform X1 [*Acyrthosiphon pisum*]	-
g41996	2.18	1.16E-04	-	-
g42342	-1	2.27E-02	-	-
g46844	2.14	2.78E-02	LOC106664738 [*Cimex lectularius*]	-
g47705	1.54	8.70E-06	Ninjurin-2-like isoform X2 [*Halyomorpha halys*]	-
g48160	-2.92	2.31E-02	TcasGA2_TC000033 [*Tribolium castaneum*]	FOG: Reverse transcriptase
g48810	2.71	3.91E-03	RNA-directed DNA polymerase from transposon X-element [*Exaiptasia pallida*]	FOG: Reverse transcriptase
g50002	1.78	1.62E-15	ADP,ATP carrier protein 2-like [*Diaphorina citri*]	Mitochondrial ADP/ATP carrier proteins

g51188	-1.32	4.74E-02	A disintegrin and metalloproteinase with thrombospondin motifs 15 isoform X4 [*Halyomorpha halys*]	Disintegrin metalloproteinases with thrombospondin repeats
g54686	-3.8	7.66E-03	-	-
g59563	-1.2	2.30E-02	BRAFLDRAFT_57765 [*Branchiostoma floridae*]	FOG: Zn-finger
g6292	-3.77	1.04E-07	RR46_07295 [*Papilio xuthus*]	-
g64784	2.34	3.95E-05	-	-
g65179	-2.4	7.11E-06	-	-
g75215	-2.57	7.53E-07	Midasin [*Bombus impatiens*]	AAA ATPase containing von Willebrand factor type A (vWA) domain
g75912	2.11	2.24E-04	Transient-receptor-potential-like protein [*Papilio polytes*]	Receptor-activated Ca2+-permeable cation channels (STRPC family)
g82369	3.3	1.95E-03	Fatty acyl-CoA reductase CG5065-like isoform X2 [Apis dorsata]	Acyl-CoA reductase
g86062	-3.12	2.12E-04	-	-
g86484	1.22	8.41E-05	Malate dehydrogenase, mitochondrial [*Nasonia vitripennis*]	NAD-dependent malate dehydrogenase
g87277	3.61	3.51E-02	D(1)-like dopamine receptor-like [*Saccoglossus kowalevskii*]	FOG: Reverse transcriptase
g87789	-4.29	1.64E-04	TcasGA2_TC031726 [*Tribolium castaneum*]	-
g89284	3.42	3.43E-02	RNA-binding protein squid-like isoform X2 [*Hyalella azteca*]	Large RNA-binding protein (RRM superfamily)

g89797	-2.87	6.91E-05	CXXC-type zinc finger protein 1-like [*Megachile rotundata*]	Uncharacterized PHD Zn-finger protein
g89907	1.46	9.17E-08	Phosphate carrier protein, mitochondrial [*Dufourea novaeangliae*]	Mitochondrial phosphate carrier protein
g91067	1.3	3.52E-02	-	-

Table S3. Top 20 highly or lowly DEGs in different *M. anisopliae* infection.

Treatment	Gene ID	Fold change (log2)	P-value	Down/Up regulated	Description
T-24h vs Blank	g51856	-8.46	1.54E-04	Down	Dvir_GJ12963
	g89208	-7.70	5.33E-04	Down	CG1732
	g32190	-7.42	5.31E-04	Down	N/A
	g71166	-7.36	3.75E-03	Down	N/A
	g53545	-7.17	5.95E-05	Down	Cuticular protein 58, RR-2 family
	g79132	-7.04	1.82E-03	Down	Dpse_GA24754
	g51258	-7.04	4.83E-04	Down	N/A
	g51851	-6.89	4.85E-03	Down	Cuticular protein RR-2 motif 77 precursor
	g12691	-6.81	1.19E-03	Down	LOC107226440 isoform X1
	g18673	-6.74	2.03E-03	Down	N/A
	g71170	-6.73	6.46E-03	Down	N/A
	g17768	-6.64	1.19E-03	Down	Nuclear factor of activated T-cells 5 isoform X3
	g74330	-6.59	6.83E-05	Down	N/A
	g16831	-6.32	7.04E-06	Down	N/A
	g17771	-6.21	1.62E-03	Down	LOC105555728
	g80315	-6.11	2.53E-02	Down	Elongation of very long chain fatty acids protein
	g17767	-6.09	1.72E-03	Down	Predicted protein
	g4624	-6.08	6.36E-03	Down	Ejaculatory bulb-specific protein 3-like
	g39720	-6.07	1.36E-03	Down	Nose resistant to fluoxetine protein 6-like
	g85623	-6.06	1.66E-03	Down	LOC103317810
	g39683	6.09	5.26E-06	Up	Retrovirus-related Pol polyprotein from transposon 17.6 isoform X4
	g72813	5.86	3.58E-05	Up	N/A
	g78763	5.85	3.50E-12	Up	N/A

	g66461	5.71	2.69E-03	Up	N/A
	g6565	5.67	2.19E-05	Up	Hypothetical protein LOTGIDRAFT_155849
	g72427	5.46	5.31E-04	Up	N/A
	g48188	5.38	2.44E-04	Up	LOC108374527
	g33838	5.28	4.22E-02	Up	N/A
	g27598	5.27	1.45E-03	Up	N/A
	g63890	5.11	1.93E-02	Up	Hypothetical protein T265_07045
	g25215	5.00	2.20E-03	Up	N/A
	g48732	4.98	6.62E-03	Up	N/A
	g85337	4.93	6.64E-03	Up	N/A
	g54345	4.90	1.59E-02	Up	Putative RNA-directed DNA polymerase
	g4457	4.89	2.05E-02	Up	N/A
	g11837	4.83	1.63E-02	Up	Pol protein
	g72815	4.75	5.33E-03	Up	N/A
	g8624	4.72	1.41E-02	Up	N/A
	g68430	4.72	1.41E-02	Up	LOC107358461
	g36211	4.71	7.52E-04	Up	RNA-directed DNA polymerase from mobile element jockey
	g23323	4.71	1.43E-02	Up	RNA-directed DNA polymerase from mobile element jockey
T-24h vs CK-24h	g75799	-10.13	1.28E-09	Down	N/A
	g32190	-9.94	4.23E-36	Down	N/A
	g75856	-9.42	9.26E-03	Down	N/A
	g89208	-9.41	1.22E-24	Down	N/A
	g89906	-9.11	5.02E-06	Down	vitellogenin
	g51258	-9.10	1.18E-25	Down	N/A
	g12691	-8.33	1.59E-49	Down	LOC107226440 isoform X1
	g85623	-7.95	1.74E-23	Down	LOC103317810
	g89900	-7.93	4.21E-05	Down	vitellogenin
	g85626	-7.89	2.32E-16	Down	LOC106786168, partial
	g51259	-7.84	3.86E-08	Down	N/A

g89852	-7.82	2.26E-23	Down	Serine protease
g36224	-7.68	3.74E-122	Down	N/A
g46261	-7.65	1.06E-20	Down	N/A
g28615	-7.56	9.43E-54	Down	Farnesol dehydrogenase
g17768	-7.50	2.31E-24	Down	Nuclear factor of activated T-cells 5 isoform X3
g17771	-7.48	1.31E-08	Down	LOC105555728, partial
g75800	-7.44	1.40E-07	Down	N/A
g89903	-7.43	7.79E-04	Down	vitellogenin-1-like
g17767	-7.37	7.96E-14	Down	N/A
g36211	5.95	1.95E-04	Up	RNA-directed DNA polymerase from mobile element jockey-like isoform X1
g20540	5.48	2.50E-04	Up	LOC107228190
g68132	5.42	1.69E-03	Up	RNA-directed DNA polymerase from mobile element jockey
g27842	4.83	1.65E-04	Up	N/A
g11366	4.82	6.43E-05	Up	N/A
g31405	4.81	4.15E-02	Up	RNA-directed DNA polymerase from mobile element jockey
g55041	4.78	8.15E-03	Up	N/A
g73578	4.70	1.40E-02	Up	N/A
Novel00465	4.61	1.89E-02	Up	N/A
g87480	4.58	4.96E-02	Up	RNA-directed DNA polymerase (reverse transcriptase) domain containing protein
g54809	4.56	4.53E-02	Up	OCBIM_22021513mg
g4893	4.53	2.46E-02	Up	N/A
g40309	4.43	4.47E-02	Up	N/A
g62062	4.40	4.26E-02	Up	LOC108368960
g67837	4.38	2.51E-02	Up	N/A
g8619	4.33	3.18E-02	Up	N/A

	g50341	4.28	3.33E-02	Up	N/A
	g4305	4.24	4.45E-02	Up	LOC106083336
	g20130	4.18	4.37E-02	Up	Hypothetical protein EAG_00403, partial
	g64596	4.12	3.57E-02	Up	N/A
	g485	3.91	1.38E-02	Up	N/A
T-48h vs Blank	g71170	-8.86	1.71E-03	Down	N/A
	g53545	-8.51	1.68E-05	Down	Cuticular protein 58, RR-2 family
	g16840	-8.15	1.07E-33	Down	Pro-resilin
	g74330	-7.33	1.92E-05	Down	N/A
	g39720	-7.13	4.41E-04	Down	Nose resistant to fluoxetine protein 6-like
	g66806	-6.79	8.04E-05	Down	Cytochrome P450
	g4624	-6.72	3.95E-03	Down	Ejaculatory bulb-specific protein 3-like
	g59286	-6.67	5.06E-03	Down	Nose resistant to fluoxetine protein 6
	g78005	-6.67	1.69E-11	Down	LOC108556711 isoform X2
	g50614	-6.67	1.24E-08	Down	N/A
	g24016	-6.61	1.18E-08	Down	GM13288
	g69034	-6.52	1.23E-03	Down	Cytochrome P450 4V2
	g66804	-6.47	3.27E-04	Down	Cytochrome P450 18a1
	g7406	-6.42	3.22E-06	Down	Organic cation transporter protein
	g15861	-6.20	8.20E-18	Down	Cuticle protein 7
	g65004	-6.19	3.33E-08	Down	LOC105666492 isoform X1
	g88916	-6.05	3.32E-07	Down	LOC106661408 isoform X2
	g12362	-6.03	2.86E-03	Down	Protein yellow-like
	g30519	-6.02	3.12E-08	Down	N/A
	g30535	-6.00	7.95E-11	Down	N/A
	g79132	-5.97	5.91E-03	Down	Dpse_GA24754

	g62949	11.73	6.10E-05	Up	N/A
	g75856	10.61	3.74E-04	Up	N/A
	g89903	9.58	2.14E-03	Up	Vitellogenin-1-like
	g89906	8.93	5.18E-05	Up	Vitellogenin
	g89900	8.64	8.80E-05	Up	Vitellogenin
	g28973	8.13	2.06E-04	Up	N/A
	g29034	8.12	7.87E-03	Up	Protein O-linked-mannose beta-1,2-N-acetylglucosaminyltransferase 1
	g15379	7.19	1.53E-03	Up	Multiple inositol polyphosphate phosphatase 1-like isoform X2
	g45861	6.65	1.00E-03	Up	N/A
	g15378	6.43	1.34E-03	Up	Nultiple inositol polyphosphate phosphatase 1
	g31049	6.08	2.22E-03	Up	N/A
	g82336	6.04	8.82E-03	Up	N/A
	g50487	5.97	2.00E-04	Up	Odorant receptor 43a-2
	g63605	5.85	6.20E-04	Up	LOTGIDRAFT_212414
	g46020	5.77	1.50E-02	Up	N/A
	g37859	5.71	1.27E-02	Up	N/A
	g85812	5.63	1.48E-03	Up	Vitellogenin 1
	g37858	5.44	9.38E-03	Up	N/A
	g39683	5.41	1.48E-03	Up	Petrovirus-related Pol polyprotein
T-48h vs CK-48h	g44681	-11.12	3.47E-02	Down	N/A
	g29497	-5.59	5.98E-03	Down	Hypothetical protein
	g13833	-4.84	1.06E-02	Down	LOC108364196
	g67454	-4.58	2.48E-02	Down	N/A
	g48675	-4.42	4.29E-02	Down	Nucleic-acid-binding protein
	g85561	-4.25	1.67E-02	Down	N/A
	g16176	-4.22	4.15E-02	Down	CAPTEDRAFT_91439, partial

g40913	-4.21	4.23E-02	Down	RNA-directed DNA polymerase from mobile element jockey
g10305	-4.03	1.23E-02	Down	LOC100680303
g40442	-4.01	7.80E-03	Down	LOC108253938
g85579	-3.63	3.21E-02	Down	N/A
g56305	-3.60	4.88E-02	Down	Cardioacceleratory peptide receptor 2 isoform X2
g5984	-3.58	1.14E-02	Down	Defensin A
g47725	-3.57	1.91E-02	Down	Dana_GF27721
g27888	-3.50	3.40E-02	Down	Reverse ribonuclease integrase
g30680	-3.27	3.45E-02	Down	PiggyBac transposable element-derived protein 4-like isoform X1
g59266	-3.24	1.74E-02	Down	Ras GTPase-activating protein 1
g58378	-3.24	3.45E-02	Down	N/A
g36683	-3.23	1.93E-02	Down	Zinc finger protein 271-like isoform X2
g30565	-3.19	6.19E-04	Down	N/A
g24308	-3.12	1.83E-02	Down	N/A
g65	-3.11	2.22E-02	Down	Retrovirus-related Pol polyprotein
g47480	6.45	3.34E-02	Up	N/A
g77554	5.90	4.75E-05	Up	N/A
g45775	5.00	2.26E-02	Up	ES protein
g21384	4.38	4.70E-02	Up	Rna-directed dna polymerase from mobile element jockey
g65450	4.36	4.10E-02	Up	N/A
g71543	4.31	1.39E-04	Up	Adult-specific cuticular protein ACP-20-like isoform X1
g57847	4.20	3.78E-02	Up	Putative RNA-directed DNA polymerase from transposon BS

g49807	4.14	3.27E-02	Up	RNA-directed DNA polymerase from mobile element jockey
g25353	4.02	4.43E-02	Up	RNA-directed DNA polymerase from mobile element jockey-like
g70401	3.99	1.17E-03	Up	N/A
g123	3.87	4.90E-04	Up	HELRODRAFT_173799
g88013	3.86	1.86E-02	Up	Hypothetical protein Y032_0290g1543
g4144	3.74	3.17E-02	Up	Hypothetical protein Y032_0258g465
g87277	3.71	1.24E-02	Up	D(1)-like dopamine receptor
g39713	3.49	3.55E-02	Up	Envelope protein
Novel00089	3.48	4.74E-02	Up	N/A
g48810	3.45	1.68E-02	Up	Putative RNA-directed DNA polymerase from transposon X-element
g24423	3.42	6.25E-03	Up	N/A
g48745	3.38	4.49E-03	Up	Dana_GF27671
g40521	3.34	3.48E-02	Up	LOC105431000 isoform X2
g11771	3.32	1.42E-02	Up	N/A
